# Chemo-manipulation of tumor blood vessels by a metal-based anticancer complex enhances antitumor therapy

**DOI:** 10.1038/s41598-018-28589-2

**Published:** 2018-07-06

**Authors:** Tina Riedel, Sabrina Cavin, Hubert van den Bergh, Thorsten Krueger, Lucas Liaudet, Hans-Beat Ris, Paul J. Dyson, Jean Y. Perentes

**Affiliations:** 1 Institute of Chemical Sciences and Engineering, Swiss Federal Institute of Technology (EPFL), 1015 Lausanne, Switzerland; 2 Service of Thoracic Surgery, Centre Hospitalier Universitaire Vaudois, 1011 Lausanne, Switzerland; 3 Service of Adult Critical Care Medicine, Centre Hospitalier Universitaire Vaudois, 1011 Lausanne, Switzerland

## Abstract

Human pleural mesothelioma is an incurable and chemoresistant cancer. Using a nude mouse xenograft model of human pleural mesothelioma, we show that RAPTA-T, a compound undergoing preclinical evaluation, enhances tumor vascular function by decreasing blood vessel tortuosity and dilation, while increasing the coverage of endothelial cells by pericytes and vessel perfusion within tumors. This in turn significantly reduces the interstitial fluid pressure and increases oxygenation in the tumor. Consequently, RAPTA-T pre-treatment followed by the application of cisplatin or liposomal cisplatin (Lipoplatin) leads to increased levels of the cytotoxin in the tumor and enhanced mesothelioma growth inhibition. We demonstrate that the vascular changes induced by RAPTA-T are related, in part, to the inhibition of poly-(ADP-ribose) polymerase 1 (PARP-1) which is associated to tumor vascular stabilization. These findings suggest novel therapeutic implications for RAPTA-T to create conditions for superior drug uptake and efficacy of approved cytotoxic anti-cancer drugs in malignant pleural mesothelioma and potentially other chemoresistant tumors.

## Introduction

Malignant pleural mesothelioma is an aggressive cancer for which curative treatments are not available at present. The disease is caused, in general, by the inhalation of asbestos fibers and develops in the pleura or peritoneum, composed of a single layer of mesothelial cells resting on a thin membrane of connective tissue, which surround the lungs and the bowel. Early-stage pleural mesothelioma can be surgically removed in the context of multimodal therapy including chemo- and/or radiation therapy, but has very high recurrence rates^1,2^. Many patients are diagnosed at an advanced stage of the disease, at which point most therapies are ineffective and surgery is only performed with a palliative intent^2^. The median survival time for patients is ≤15 months depending on the stage of disease progression at the time of diagnosis. Since 2003, the standard chemotherapy regimen for induction and palliative treatment has been the combination of cisplatin with pemetrexed. While survival was proven to be enhanced, the median time to progression was only of 5.7 months^3,4^. Despite many attempts, until now, no regimen has further improved survival.

There is increasing evidence that efficacy of chemotherapy is often limited by insufficient uptake and distribution in tumors. Anticancer drugs must reach target tumor cells through blood vessels and then penetrate through the tumor tissue, a mechanism that relies on diffusion and convection. One barrier for efficient drug delivery in solid tumors is the morphologically and functionally abnormal tumor vasculature characterized by chaotic angiogenesis and highly permeable blood vessels that lead to elevated tumor interstitial fluid pressure^5^. High interstitial fluid pressure is thought to impede convection and, for this reason, to cause unfavorable drug transport conditions within tumors. The latter is, in turn, believed to be responsible for the poor response of tumors to chemotherapy and their recurrence^6^. Ways to improve tumor vasculature and drug transport have since been described. *Jain et al*. first demonstrated that anti-angiogenic therapy at low doses could normalize the vasculature of solid tumors leaving behind a more mature and functional vessel network by selectively pruning immature blood vessels^7,8^. This process enhances tumor oxygenation and drug transport, thus improving the effect of radiation- and chemotherapy^7,8^. Vascular normalization with VEGF and VEGFR inhibitors has been demonstrated in several preclinical cancer models^7,8^. Recently, the normalization concept was also confirmed in patients with colorectal and brain tumors^9,10^. However, the application of anti-angiogenic therapies to achieve vascular normalization requires a fine-tuned dose control; a too small dose has no effect whereas a too high dose prunes away all vessels. In addition, anti-angiogenic treatments are notorious for their side effects on wound healing, blood pressure modulations and kidney toxicity^10^. Thus, the clinical translation of vascular normalization has remained difficult in patients. Other approaches are currently being studied to optimize vascular normalization while avoiding side effects, including more specific vascular modulating agents such as tyrosine kinase inhibitors^11^, low dose photodynamic therapy approaches^12,13^ and others^14^.

**Figure 1:**
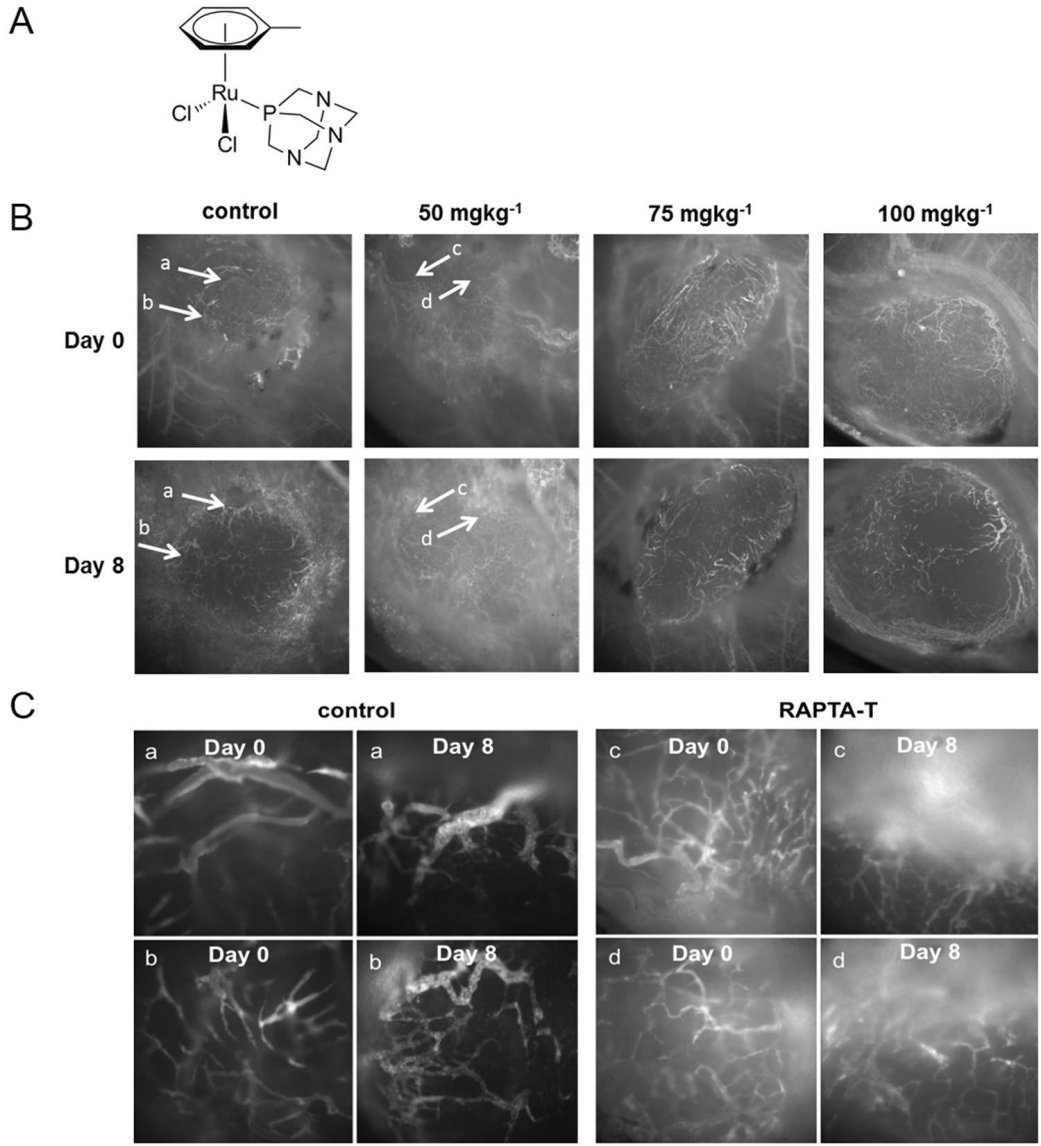
Real time assessment of MSTO-211H tumor and vascular changes following RAPTA-T treatment. (**A**) Chemical structure of RAPTA-T (Ru(η^6^-toluene)(PTA)Cl_2_). (**B**) Intravital microscopy images at days 0 and 8 show the progressive vascular changes upon daily RAPTA-T (50, 75, 100 mg kg^−1^) treatment. White arrows point to the vessels that were traced during RAPTA-T treatment (50 mg kg^−1^) over 8 days in (**C**). (**C**) Regions with the same vessels (a–d) at day 0 and day 8 in (A) are magnified 20× . Vessels within control tumors at day 0 and at day 8 were chaotic and dilated with abrupt changes in vessel diameter and direction. Vessels in treated tumors day 0 appeared similar to control tumors on day 0 but had a less dilated and more “normal” structural phenotype at day 8.

Recently, a new class of promising organometallic anticancer agents (termed RAPTA, Fig. 1A) has been developed which exhibit a broad spectrum of activity including effects on resistance and sensitizing phenomena of cancer cells, but with surprisingly low general cytotoxicity. In preclinical cancer models, RAPTA compounds inhibit primary tumor growth and the development of metastases^15–17^. In addition to their anticancer properties these organometallics display anti-angiogenic activity *in vivo*, although the effect at even high concentrations was relatively mild in comparison to other anti-angiogenic compounds^18^.

Based on the moderate anti-angiogenic activity and low *in vitro* cytotoxicity on cancer and endothelial cells, we hypothesized that these drug properties would be desirable and could be explored to favor vascular normalization without risking too high anti-angiogenic activity and excessive vessel pruning. With this objective, we assessed the impact of RAPTA-T [Ru(η^6^-toluene)(PTA)Cl_2_] treatment on the tumor vascular morphology and function in a murine pleural mesothelioma model by real time imaging. We show that RAPTA-T, at a low dose, normalizes tumor blood vessels and increases oxygenation through improved vascular function. By combining RAPTA-T with cisplatin, one of the first-line chemotherapeutic agents for mesothelioma, we demonstrate improved treatment outcome through higher chemotherapeutic drug uptake into the tumor. Finally, we demonstrate that RAPTA-T can inhibit Poly(ADP-Ribose) Polymerase type 1 (PARP-1), a nuclear protein involved in DNA damage repair and angiogenesis. We show that PARP-1 inhibition is associated with a drop in vascular endothelial growth factor secretion *in vitro* and promotes vascular stabilization *in vivo*. These results suggest a chemotherapeutic combination therapy with RAPTA-T as a novel option in resectable (as neoadjuvant therapy) and advanced (as palliative therapy) mesothelioma or for the management of other solid tumor cancer types.

## Results

### RAPTA-T inhibits tumor Poly(ADP-Ribose) Polymerase type 1 *in vitro* which decreases tumor derived vascular endothelial growth factor (VEGF)

As a metal-based compound, RAPTA-T has the capacity to dislodge zinc ions from zinc finger proteins and change their conformation and function^19^. We first determined the impact of RAPTA-T on PARP-1 in an *in vitro* colorimetric assay. We found that PARP-1 activity was inhibited by RAPTA with an IC_50_ of 400 μM (Fig S1A). PARP-1 is activated in response to DNA damage induced by various genotoxic agents such as the oxidant H_2_O_2_. In turn activated PARP-1 induces PARylation of multiple proteins and promotes cell necrosis through ATP depletion. To explore whether RAPTA-T could interfere with such processes, we cultured MSTO211H cells in the presence of H_2_O_2_ (250 µM), with or without pretreatment with RAPTA-T (400 μM), or 3AB (a prototypical PARP-1 inhibitor, 150 µM). The H_2_O_2_ cytotoxicity was significantly and comparably reduced by 3-AB and RAPTA-T (Fig S1B). Furthermore, the cytoprotective effects of 3-AB and RAPTA-T were associated with a trend to a decreased accumulation of PAR polymers in cells exposed to H_2_O_2_ (Fig S1C). It is also noteworthy that PARP-1 has been shown to be involved in the synthesis of vascular endothelial growth factor (VEGF)^20,21^. We therefore determined the impact of RAPTA-T on VEGF released by MSTO211H cells in culture, and we found that RAPTA-T (400 μM) caused a two-fold drop in VEGF-A levels (Fig S1D). Taken together, these results support the notion that RAPTA-T behaves as an inhibitor of PARP-1 activity.

### RAPTA-T alters the tumor vascular morphology *in vivo*

RAPTA-T has been shown to have no cytotoxic effect on vascular endothelial cells^18^. Using a cell viability assay, we excluded a direct cytotoxic effect of increasing doses of RAPTA-T on cancer cells (Fig S2). We then investigated the effect of RAPTA-T on mesothelioma xenografts in real time by assessing the vascular changes of MSTO-211H tumors grown in dorsal skinfold chambers using fluorescence intravital microscopy. The dorsal skinfold chamber is a sophisticated window model that allows detailed observation of the effects of a drug on tumor blood vessels over multiple days in a non-invasive manner. Functional blood vessels are visualized by injecting a high-molecular weight fluorescent molecule (FITC-dextran 2,000 kDa) intravenously prior to imaging. Tumors were treated by daily RAPTA-T doses (50, 75 and 100 mg kg^−1^) for a maximal period of 18 days. None of the RAPTA-T concentrations caused tumor shrinkage (Fig. 1B). Progressive tumor vascular changes with increasing doses were observed. Low dose RAPTA-T (50 mg kg^−1^) decreased tumor vascular dilation and tortuosity by day 8 compared to controls (Fig. 1C). These characteristics persisted until day 11, but shifted towards vessel regression by day 18 (Fig S3). Higher RAPTA-T doses (75 and 100 mg kg^−1^) caused similar changes but also caused vascular pruning effects by day 8 (Fig. 1B, Fig S3). In contrast, the vasculature of healthy tissue remained unchanged during the course of treatment at all concentrations. Interestingly, while low dose RAPTA-T was tolerated by animals, higher doses caused weight loss and spontaneous deaths over the course of treatment. Based on the observed vascular morphology changes and toxicity upon RAPTA-T treatment, we estimated an appropriate treatment window for RAPTA-T at a tolerated daily dose of 50 mg kg^−1^ for a minimum of 8 days and a maximum of 11 days.

**Figure 2:**
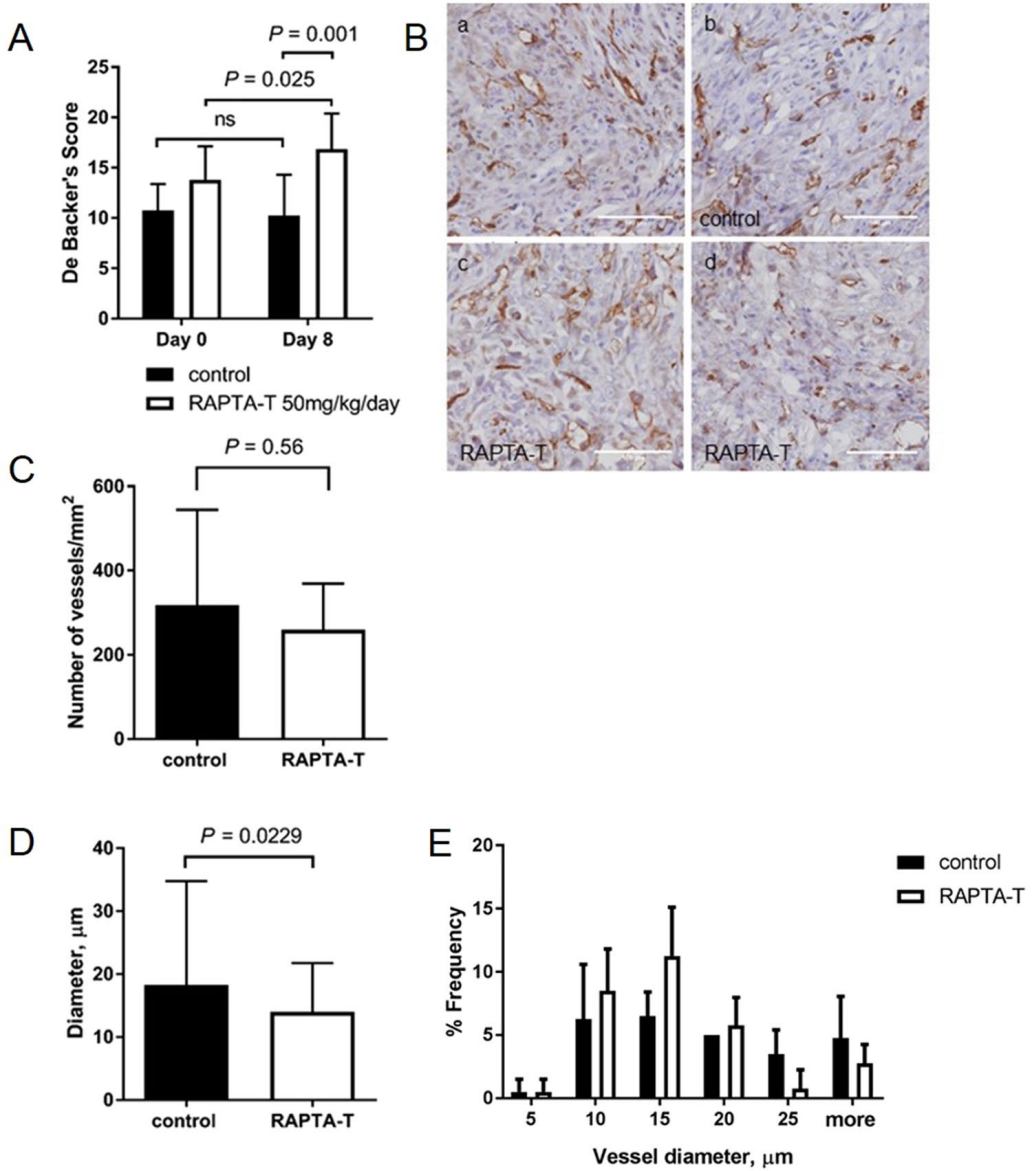
Change of vessel density and diameter following 8 and 10 days of 50 mg kg^−1^ RAPTA-T treatment. (**A**) Quantitiation of tumor vessel density in dorsal skinfold chambers by the DeBacker method after 8 days of treatment. Vessel density slightly increases from day 0 to day 8 in RAPTA-T treated tumors (*N* = 3, DeBacker’s score 13.7 ± 3.3 at day 0 and 17.1 ± 3.5 at day 8, *P* = 0.025, 2-way ANOVA), but not in controls (*N* = 4, DeBacker’s score 10.8 ± 2.6 at day 0 and 10.2 ± 4.1 at day 8, *P* = 0.63, 2-way ANOVA). Data represent mean ± SD. (**B**) Representative brightfield tumor sections stained for CD31 (magnification x40) after 10 days of treatment. (**C**) Microvessel density (MVD) was counted as number of vessels per mm^2^ in controls (*N* = 4, a and c) and RAPTA-T treated tumors (*N* = 5, b and d). The mean vascular density was 318 ± 226 for control tumors and 260 ± 109 vessels/mm^2^ for RAPTA-T treated tumors, respectively, after 10 days of treatment. Data represent mean ± SD. (*P* = 0.56, unpaired *t*-test, Mann-Whitney). (**D**) Mean vessel diameter was significantly decreased in RAPTA-T treated tumors compared to control tumors (18.3 ± 16.4 vs 14.0 ± 7.7, respectively, *N* = 4–5 mice per group). (**E**) Percentage frequency distribution of vessel diameters shows a shift from 20 μm and larger diameter vessels in control tumors to 5–15 μm diameter vessels in RAPTA-T treated tumors (*N* = 120–140 vessels per group).

Next, the effect of daily RAPTA-T (50 mg kg^−1^) treatment over 8 days on the vascular density of tumors grown in dorsal skinfold chambers was quantified. The density of perfused vessels increased significantly in RAPTA-T treated tumors over 8 days (DeBacker’s score index 13.7 ± 3.3 at day 0 and 17.1 ± 3.5 at day 8, *P* = 0.025, 2-way ANOVA, *N* = 3), whereas in control tumors the perfused vessel density did not change over time (DeBacker’s score 10.8 ± 2.6 at day 0 and 10.2 ± 4.1 at day 8, *P* = 0.629, 2-way ANOVA, *N* = 4) (Fig. 2A). To assess the effect of RAPTA-T on the vessels throughout the depth of tumors, we quantified vessel density of tumor sections stained for CD31. Tumors were treated with daily doses of RAPTA-T (50 mg kg^−1^) over a period of 10 days. Tumor vessel diameter at day 11 was also assessed on these sections. There was no significant change in vessel density following RAPTA-T treatment compared to controls on day 11 (260 ± 109 vessels/mm^2^ vs 318 ± 226/mm^2^, respectively, *N* = 4–5 mice per group, *P* = 0.56) (Fig. 2B,C). Interestingly, tumor vessel diameter was significantly decreased in RAPTA-T tumors compared to controls (18.3 ± 16.4 μm vs 14.0 ± 7.7 μm, respectively, *N* = 4–5 mice per group, Fig. 2D), with a shift from 20 μm and larger diameter vessels to 5–15 μm diameter vessels (*N* = 120–140 vessels per group, Fig. 2E).

### RAPTA-T reduces interstitial fluid pressure (IFP), improves vascular function and transport in tumors and enhances cisplatin uptake

**Figure 3:**
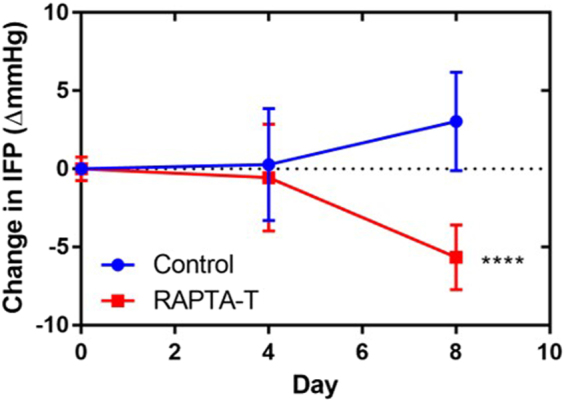
RAPTA-T 50 mg kg^−1^ lowers interstitial fluid pressure in MSTO-211H tumors. IFP measurements were performed using the wick-in-needle technique. 50 mg kg^−1^ RAPTA-T or 0.9% NaCl (aq.) as control were given daily i.p. over 8 days. The IFP value was normalized by the value at day 0. Data are represented as mean ± SD. ****P < 0.0001 (2-way ANOVA).

Abnormal blood vessel function contributes to elevated IFP in tumors, which in turn adds to a decreased transcapillary transport and impairs drug delivery throughout tumors^6^. We evaluated how daily RAPTA-T (50 mg kg^−1^) treatment affected tumor interstitial fluid pressure (IFP) in MSTO-211H tumors over a period of 8 days. Between days 0 and 4, there were no significant changes in IFP of the tumor and surrounding normal tissue between the control and RAPTA-T treated tumors. From day 5 to day 8, a significant drop in tumor IFP was observed in the RAPTA-T treated group, but not in controls (∆mmHg 0.2 ± 3.5 on day 4 and −5.6 ± 2.07 on day 8 for RAPTA-T treated group (*P* = 0.003) vs ∆mmHg 0.3 ± 3.6 on day 4 and 3.0 ± 3.2 on day 8 for control group (*P* = 0.19). At day 8, RAPTA-T treated tumors had a significantly lower IFP compared to controls (5.7 ± 3.5 vs 13.2 ± 1.1 mmHg, *N* = 6–7 mice per group, *P* < 0.0001 2-way ANOVA, Fig. 3).

**Figure 4:**
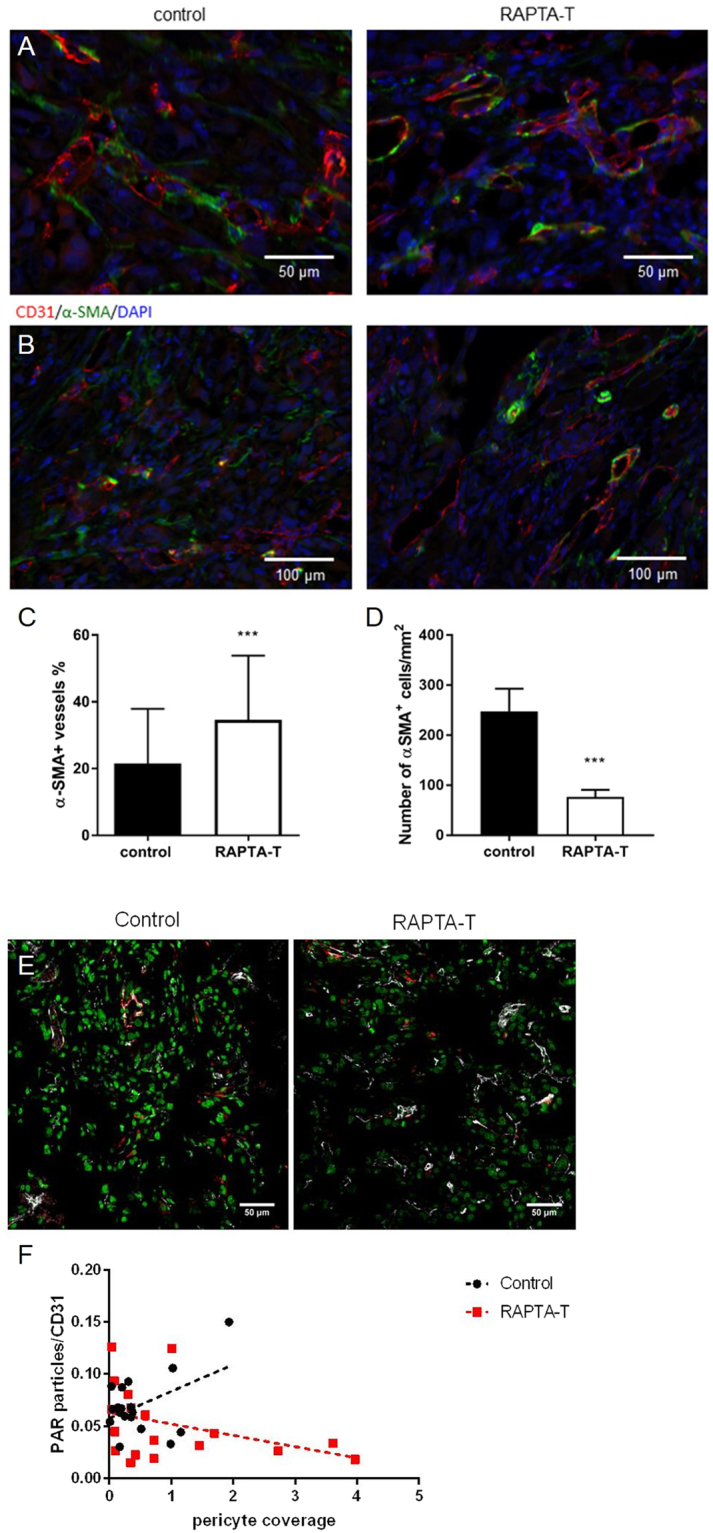
RAPTA-T treatment improves vascular pericyte coverage of tumor vessels. (**A**) Pericyte coverage was determined in MSTO-211H tumors treated with vehicle (left panels) or daily 50 mg kg^−1^ RAPTA-T (right panels) for 10 days. Immunohistochemical double stainings of CD31 and α-SMA show that in control tumors α-SMA positive pericytes were partially detached from the vessels (red). Arrows are pointing at loosely attached pericytes. In RAPTA-T treated tumors tighter contacts between pericytes and endothelial cells were observed. (**B**) Double staining of CD31 (red) and α-SMA (green) shows significantly more pericyte-covered tumor vessels in RAPTA-T treated (right panel) than control MSTO-211H tumors (left panel), quantified in (**C**). ***P < 0.001 (unpaired *t*-test with Welch’s correction) (**D**) Quantification of α-SMA positive cells, which are not associated to endothelial cells, representing cancer-associated fibroblasts. Mean ± SEM, ***P < 0.001 (unpaired *t*-test with Welch’s correction). Fluorescent images were captured at 20x magnification. (**E**) Triple immunostaining for PAR (green), CD31 (white) and alpha-SMA (red) in heterotopic MSTO211H tumors. (**F**) The graph represents the quantification of PAR+ particles in the surrounding of tumor vessels as a function of the vascular pericyte coverage (N = 5–4/group, 18–19 fields/group). In RAPTA-T treated tumors, an inverted linear correlation between PAR expression and pericyte coverage enhancement was observed.

To determine whether RAPTA-T modulated the vascular wall structure, we assessed tumor vascular pericyte coverage by double-immunostaining of the endothelial cell marker CD31 and the pericyte marker α-SMA. On day 11, we found more α-SMA-positive pericytes that were partially detached from the vessels in control tumors compared to RAPTA-T treated tumors, where tighter contacts between pericytes and endothelial cells were observed (Fig. 4A). Pericyte coverage was significantly enhanced in RAPTA-T (50 mg kg^−1^) treated tumors compared to controls (34.6 ± 19.2 vs 21.5 ± 16.3% α-SMA-positive vessels, respectively, Fig. 4A,C, *P* < 0.001, unpaired *t*-test). Also, the endothelial cell lining was more homogeneous in RAPTA-T treated tumors compared to controls. In addition, there were significantly less α-SMA-positive stained cells away from the vessel perimeter in RAPTA-T treated tumors compared to controls (***P < 0.001 unpaired t-test with Welch’s correction, Fig. 4B,D).

Based on our *in vitro* findings where RAPTA-T could lead to PARP-1 inhibition, we determined how tumor derived PAR levels correlated with vascular pericyte coverage by immunohistochemistry *in vivo*. We found that enhanced pericyte covage in the RAPTA-T treated tumors correlated with a drop in tumor derived PAR (linear correlation), while this was not the case in the control tumors (Fig. 4E,F).

**Figure 5:**
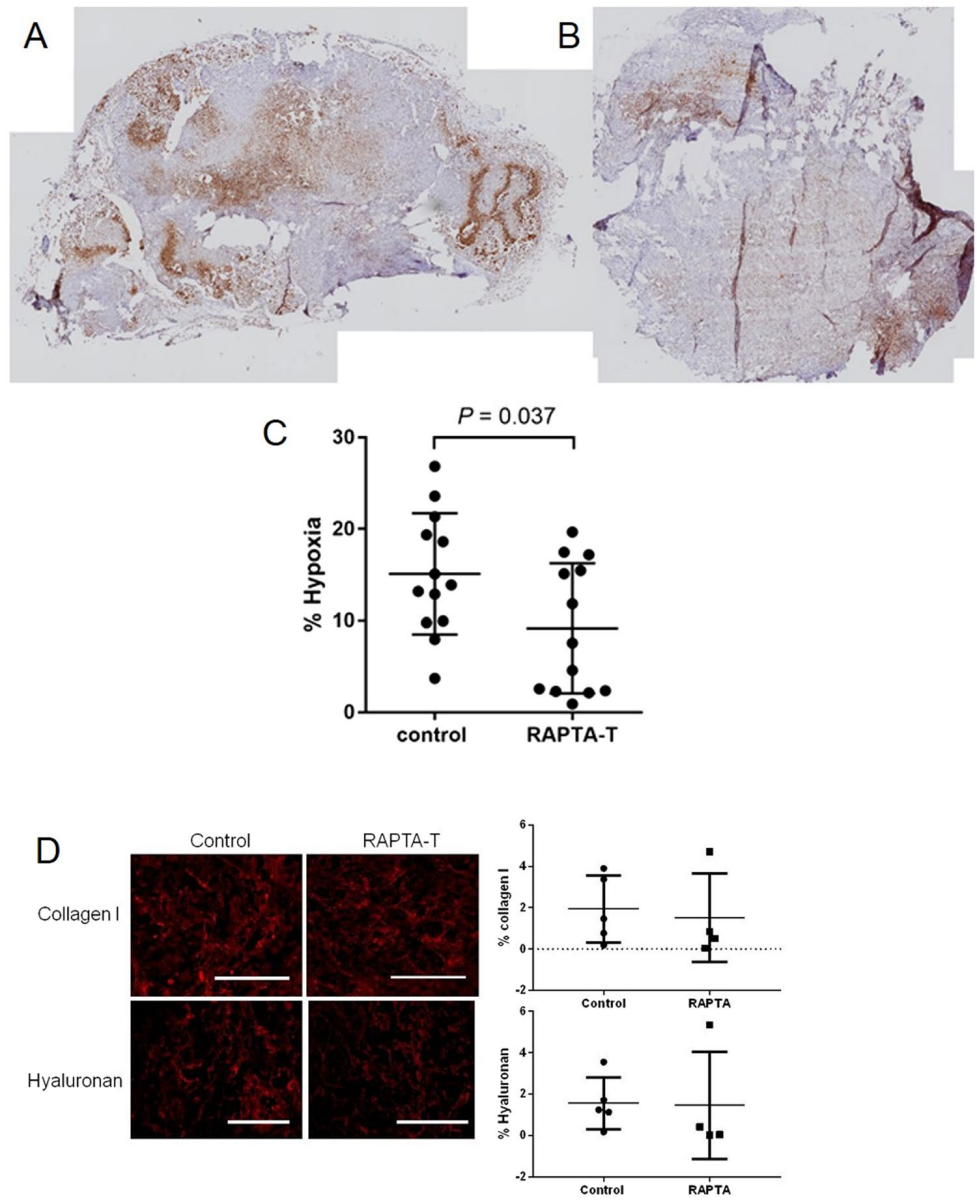
RAPTA-T treatment reduces tumor hypoxia. Immunohistochemical detection of tumor hypoxia with the exogenous 2-nitroimidazole hypoxia marker pimonidazole hydrochloride after daily RAPTA-T (50 mg kg^−1^) treatment for 10 days. Tumor sections of (**A**) control tumors and (**B**) treated tumors at day 11 were compared, and quantified in (**C**). After RAPTA-T treatment, tumor hypoxia was reduced significantly (*N* = 3 mice per group, *P* = 0.037, unpaired *t*-test). Data are represented as mean ± SD. (**D**) Collagen I and Hyaluronic acid content in MSTO211H tumors treated by RAPTA-T or control. The quantification does not reveal any significant difference in heterotopic tumors.

Given the properties of RAPTA-T to correct morphological and functional vessel abnormalities shown by improved vascular pericyte coverage and reduced IFP, blood flow in tumor vessels would be expected to be improved after treatment, which in turn would improve tumor oxygenation and hence reduce hypoxia. According to this hypothesis, we found that daily RAPTA-T treatment over 10 consecutive days significantly decreased tumor hypoxia by day 11 compared to controls (Fig. 5A–C). Also, the spatial distribution of the pimonidazole stain suggested that hypoxia was mostly decreased in the central region of tumors with RAPTA-T treatment. In parallel, we also determined the overall extracellular matrix changes in RAPTA-T treated and control tumors. RAPTA-T therapy did not cause changes in the Collagen I and hyaluronic acid content of mesothelioma tumors compared to controls in heterotopic and orthotopic tumors (Fig. 5D and Fig S4A).

**Figure 6:**
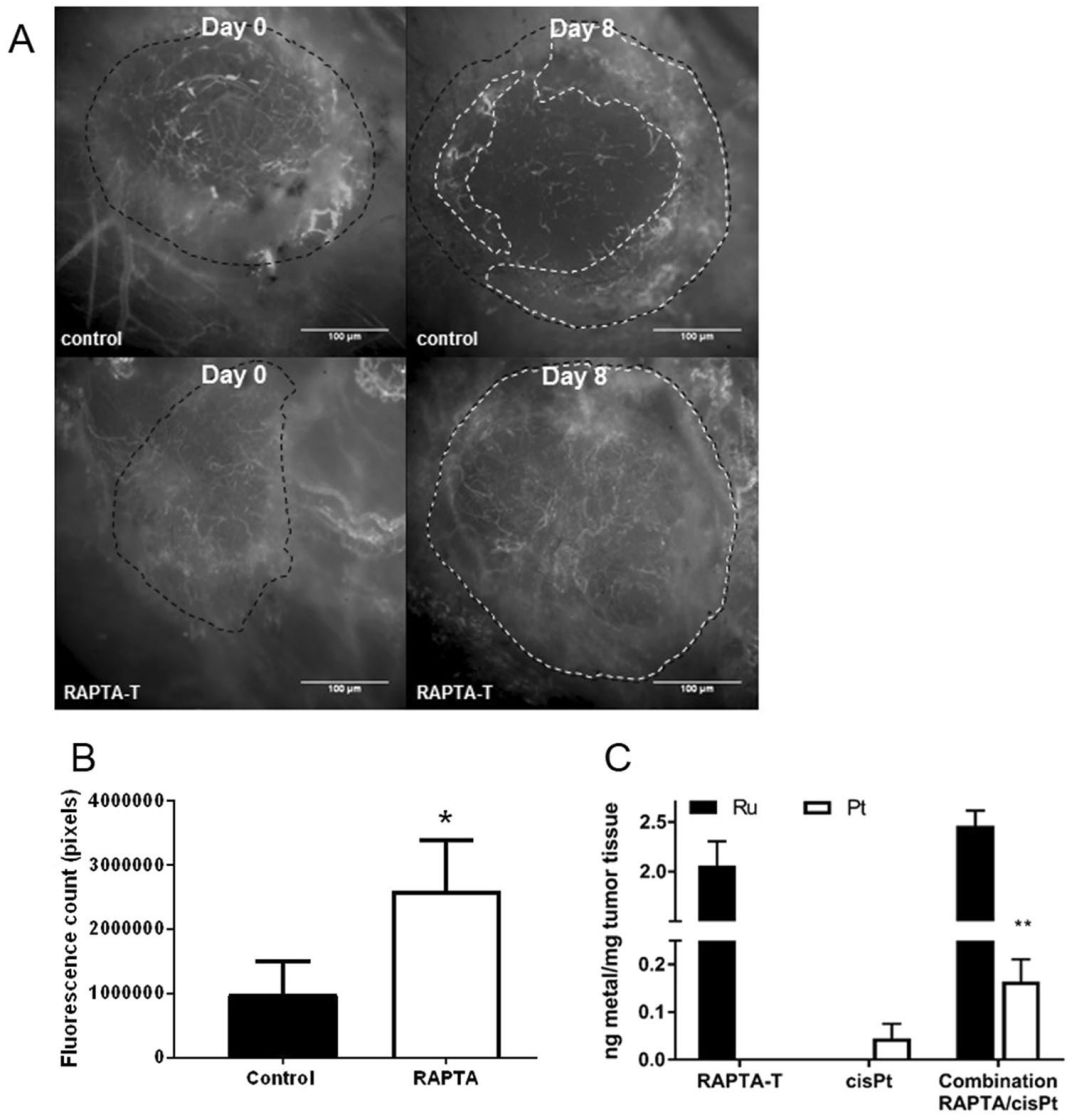
RAPTA-T pre-treatment improves vascular transport in tumors. (**A**) The effect of RAPTA-T on tumor perfusion in established MSTO-211H mesothelioma tumors (black dotted lines) in dorsal skinfold chambers was determined by measuring the distribution of a high molecular weight FITC-dextran (2,000 kDa) tracer (white dotted lines). (**B**) Within RAPTA-T treated tumors there was significantly more FITC-dextran extravasation with a more homogenous fluorescence signal throughout the tumor mass at day 8 compared with controls. (**C**) MSTO-211H tumors of mice (*N* = 4 per group) treated with RAPTA-T alone, cisplatin alone or the RAPTA/cisplatin combination were each analysed by ICP-MS for their Ruthenium (Ru) and Platinum (Pl) metal content. Mice treated with RAPTA-T or cisplatin alone had sole detection of Ru and Pl respectively (columns 1–2). Mice pre-treated with 50 mg kg^−1^ RAPTA-T showed an increased uptake of cisplatin into tumor tissue compared to mice treated with cisplatin alone but no difference in the Ru content (**P < 0.005, unpaired *t*-test). Data are represented as mean ± SD.

We then assessed how vascular modulation by RAPTA-T affected the transport of a macromolecular fluorescent dye (FITC-dextran 2,000 kDa). FITC-dextran was more homogeneously distributed and accumulated significantly more throughout tumors following 8 consecutive days of RAPTA-T treatment compared to controls (Fig. 6A,B). Again, the central core of the tumors showed most of the enhanced tracer distribution in RAPTA-T treated tumors, whereas in control tumors fluorescence was mainly confined to the tumor periphery (Fig. 6A). These results are supported by the quantitative analysis of vessel density on intravital microscopy images using the DeBacker method which show a higher density of perfused vessels at day 8 in RAPTA-T treated tumors compared to controls (*P* = 0.001, unpaired *t*-test, Fig. 2A).

To determine if the enhanced vascular transport of the fluorescent tracer translated into better drug uptake, we combined 10 days of RAPTA-T treatment with a single cisplatin dose on day 11 in MSTO-211H mesothelioma xenografts and quantified the cisplatin concentration in tumor tissues by inductively coupled plasma mass-spectrometry at day 21. RAPTA-T pre-treatment significantly enhanced the platinum concentrations in tumors (0.16 ± 0.05 ng Pt/mg tumor tissue) compared to controls (0.05 ± 0.03 ng Pt/mg tumor tissue, *P* = 0.005). The quantities of residual ruthenium were measured 11 days after pre-treatment and were comparable between the RAPTA-T (2.06 ± 0.42 ng/mg tumor tissue) and RAPTA-T + cisplatin combination group (246 ± 0.31 ng/mg tumor tissue, Fig. 6C). Interestingly, while RAPTA-T treatment alone had no significant effect on tumor growth by day 21 compared to control, there was a significant decrease in tumor growth of the RAPTA-T combination with cisplatin compared to cisplatin alone (333 ± 88 vs 164 ± 65 mm^3^ at day 21, respectively, p < 0.005, Fig S5). Taken together, these findings suggest that improvements in vascular function through RAPTA-T pre-treatment enhance the molecular transport of cisplatin across the tumor vasculature to reach target cancer cells.

### RAPTA-T pre-treatment improves efficacy of systemically administered lipoplatin in an orthotopic mesothelioma tumor model

**Figure 7:**
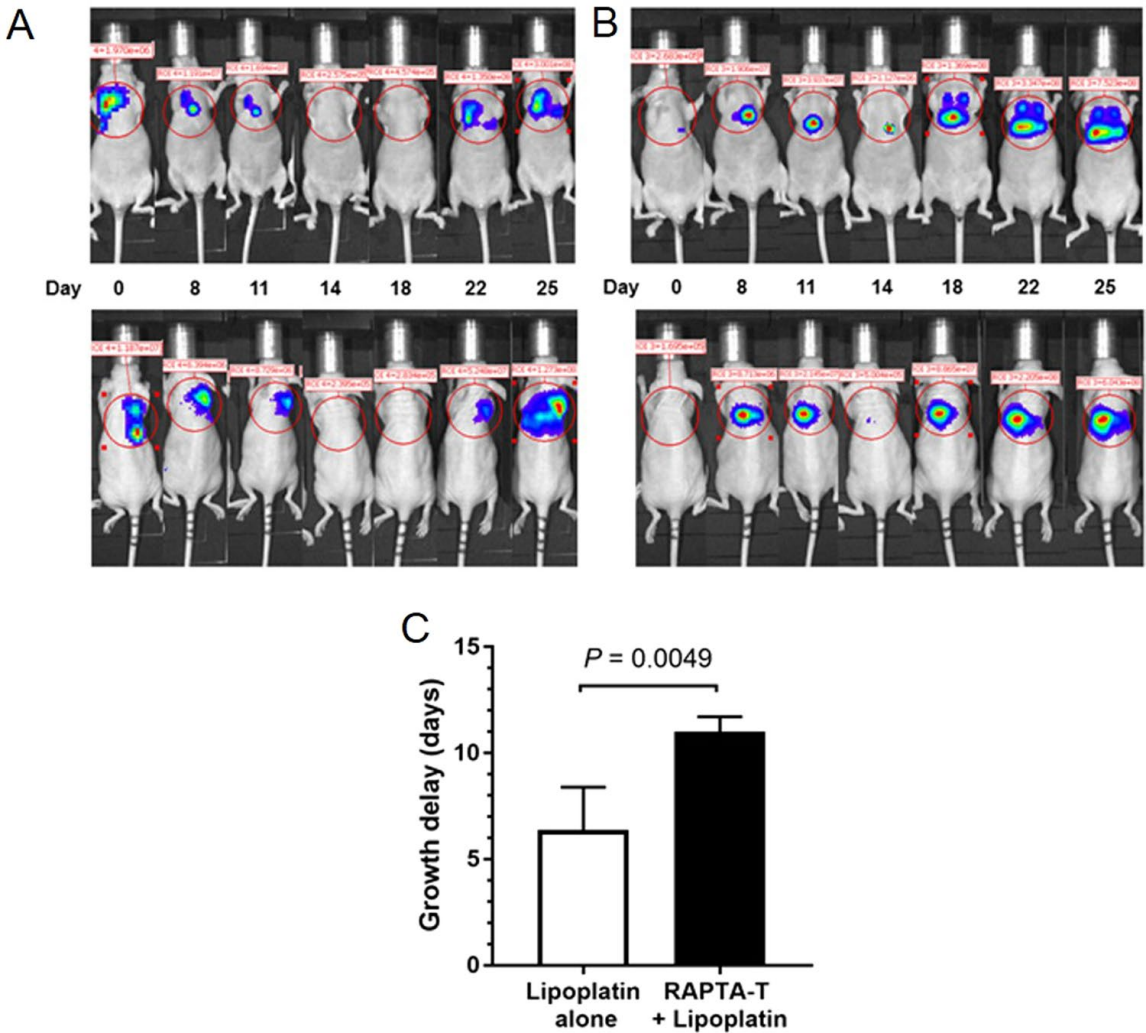
RAPTA-T pre-treatment enhances growth delay of subsequently applied drug lipoplatin in an orthotopic Hmeso-1 human mesothelioma xenograft model. Representative bioluminescence images following tumor growth of (**A**) RAPTA-T pre-treated and (**B**) control mouse. RAPTA-T or vehicle was given in eight subsequent injections (50 mg kg^−1^ body weight RAPTA-T or saline, i.p. every 24 hours from day 1 to 8) followed by a single injection of lipoplatin (18 mg kg^−1^) administered i.p. on day 9 to both pre-treated and control groups. The total photon flux released by luciferase-expressing Hmeso-1-tumor cells was measured after i.p. injection of D-luciferin (150 mg kg^−1^) in dorsal and ventral positions (upper and lower panel of images, respectively). (**C**) Lipoplatin leads to enhanced growth inhibition and delays tumor re-growth by 5 ± 2 days when administered after RAPTA-T pre-treatment (growth delay 11 ± 1 days with RAPTA-T pre-treatment compared to 6 ± 2 days with lipoplatin alone, *P* = 0.0049, unpaired *t*-test). Data represent the average of photon flux/s (log scale) measured from dorsal and ventral positions of mice (*N* = 4 per group) and are normalized to the photon flux before lipoplatin administration (day −1) and given as mean ± SD.

We next evaluated how the alterations in tumor vessel structure and function through pre-treatment with RAPTA-T affected the impact of lipoplatin chemotherapy on orthotopic pleural mesothelioma in mice. We initially confirmed that vascular stabilization under the form of enhanced pericyte coverage occurred in MSTO211H tumors (Fig S4B,C). Nude mice with established orthotopic H-meso-1 pleural mesothelioma were given daily doses of RAPTA-T (50 mg kg^−1^) or 0.9% NaCl (aq.) as the control over a period of 8 days (21 days after tumor cell inoculation). Subsequently, these mice received a single i.p. dose of lipoplatin (18 mg kg^−1^, which corresponds to one third of the LD_50_ concentration for mice (35) 24 h after the last RAPTA-T injection (on day 9), and tumor growth was followed by bioluminescence imaging (until day 29). RAPTA-T treatment had no effect on tumor growth compared to controls over the 8 day course of pre-treatment. While all mice developed tumor regression within 5 days following lipoplatin administration, RAPTA-T combined with lipoplatin caused a significantly higher growth delay compared to lipoplatin alone (growth delay of 11 ± 1 days with RAPTA-T pre-treatment compared to 6 ± 2 days with lipoplatin alone, *P* < 0.0001, Fig. 7). Also, the rate of tumor re-growth was very similar in both groups. Tumor growth curves of individual mice are shown in Fig. S6.

## Discussion

Mesothelioma is a chemoresistant cancer for which new treatment regimens and new putative drugs are urgently needed. Improved response to chemotherapy and to radiation therapy is mandatory for the management of early and more advanced mesotheliomas in the context of a multimodal therapy with curative intent or in the context of palliative therapy. In this study we have examined the possible use of the small-molecule organometallic compound RAPTA-T as a tumor vascular modulation agent in pleural mesothelioma and describe a possible mechanism of action through PARP-1 inhibition. We show the functional changes that occur result from the “remodeling” of the tumor vasculature with RAPTA-T in relevant heterotopic and orthotopic models. The abnormal structure and function of the tumor vasculature, comprising leaky and compressed vessels, leads to a microenvironment within solid tumors that is characterized by elevated IFP, inefficient tumor perfusion and focal hypoxia^7,8^. These factors impede the delivery of molecular therapeutics within tumors, induce peri-tumoral edema, impair tumor response, promote recurrence and can contribute to metastatic spread^5^. Thus, repairing an abnormal and heterogeneous microenvironment – and improving oxygen distribution in particular – can significantly improve tumor treatment including subsequent chemo- and radiation therapy and potentially immunotherapy. To the best of our knowledge, this is the first study to show that vessel modulation with an organometallic drug has functional consequences with regards to the intratumoral delivery and antitumor activity of subsequently administered chemotherapeutic drugs cisplatin and Lipoplatin.

A hallmark of solid tumors is their elevated IFP, which results from their highly permeable angiogenic vessel network as well as the absence of functional lymphatics^6^. These vascular characteristics represent barriers for therapy as they affect distribution and tumor oxygenation. Unpublished work from mesothelioma patients as well as from orthotopic models in rats has shown that mesothelioma is characterized by elevated interstitial fluid pressure (in the range of 3 to 4 mmHg)^12^. Continuous low dose administration over 10 days of RAPTA-T to mice bearing MSTO-211H xenograft tumors results in the disappearance of tortuous and dilated tumor vessels leading to a more homogeneous vascular network (Fig. 1) with smaller vessels of more uniform diameter size (Fig. 2D,E) and better perfusion (Fig. 6A). In contrast to a number of other studies^7,8,22^ concerned with anti-angiogenic therapy using VEGFR2 inhibitors or tyrosine kinase inhibitors, we found that reduced IFP (Fig. 3) is not positively correlated with a decrease in overall vessel density (Fig. 2A–C). These other studies demonstrated normalization of the tumor vasculature with a decreasing fraction of immature small tumor vessels that lack coverage by smooth muscle cells and pericytes. Consequently, a drift to higher mean tumor vessel diameters and reduced perfused vessel density was found. RAPTA-T, however, at the applied dose and window, was not found to prune vessels (Fig. 1), which is in accordance with the tumor growth rate within this apparent normalization window not being significantly inhibited (Fig. 1B and Fig S6B). Instead, RAPTA-T therapy was associated with vessel maturation leading to better transport properties (better tracer/drug distribution, better oxygenation) and lower permeability (decrease in tumor IFP).

On a cellular level, immunohistological analyses show that RAPTA-T treatment leads to an increase in the amount of microvessels covered by α-SMA+ pericytes (Fig. 4B,C). In tumors, an abnormal phenotype of α-SMA-expressing pericytes has been described^23,24^, in which pericytes are often partially detached from endothelial cells with abnormal shapes and long processes away from the vessel wall. These abnormalities have been previously described as characteristics of malfunctioning leaky vessels with wide junctions, which lead to increased IFP, and are closely linked to poor tumor perfusion and hypoxia. Our findings suggest that vascular function can be improved with RAPTA-T through the activation of pericytes and enhancement of their coverage of endothelial cells. Also, based on our *in vitro* and *in vivo* findings, RAPTA-T inhibits PARP-1, which has been shown to be important in the process of angiogenesis, possibly through modulation of MAP kinase-dependent regulation of angiogenic factor balance^20,25^. RAPTA-T-dependent PARP-1 inhibition was indeed associated with a drop in tumor cell-derived VEGF (Fig S1D). In addition, tumor cell PAR expression was significantly decreased in the areas of enhanced pericyte coverage of MSTO211H tumors grown in mice. Combined, this suggests that PARP-1 inhibition is mechanistically involved in the RAPTA-T-induced changes on the tumor vasculature. These findings are consistent with previous studied indicating that RAPTA-T can inhibit PARP activity^19^.

Based on our findings, PARP-1 was inhibited by RAPTA-T resulting in a two-fold drop in VEGF-A secretion by MSTO211H tumor cells. By tumor immunostaining analysis, we found a linear correlation between tumor derived PARP-1 expression decrease and enhanced pericyte vascular coverage in RAPTA-T treated tumors. It is well established that anti-VEGF therapy is associated to enhanced vascular pericyte coverage in tumors which decreases IFP and improves vascular transport^7,8^. Together, this suggests that PARP-1 inhibition by RAPTA-T participates in vascular stabilization through a drop in VEGF-A levels.

In addition, an effect of RAPTA-T on cancer associated-fibroblasts (CAFs), which are also known to be α-SMA immunoreactive, cannot be excluded given the lower number of α-SMA+ cells in the tumor interstitium upon treatment (Fig. 4B,D). CAFs are among the major cell populations within stromal compartments, known for their source of pro-angiogenic growth factors, extracellular matrix (ECM) remodeling proteins and production of ECM components, which mediate cancer growth and metastasis and impede efficient chemotherapeutic drug delivery^26,27^. Interestingly, ECM changes induced by a decrease in overall CAFs in tumors was not observed with collagen type I and hyaluronic acid (HA) staining, that remained comparable between RAPTA-T treated and control tumors.

Tumor vascular functionality is a contributing factor of the effectiveness of cytotoxic chemotherapy. Similar to low dose antiangiogenic therapy that has been shown to provide a short-term “normalization window” where vascular functions are improved by reducing vascular leakage and increased tumor oxygenation, 8–10 days of RAPTA-T treatment offers the benefit of enhanced antitumor effect when administered in combination with cisplatin during that time window. The combination of RAPTA-T pre-treatment with cisplatin, the standard treatment for pleural mesothelioma in the clinic, leads to a better antitumor effect in a heterotopic mouse model than either therapy alone (Fig S6B,C). Increased levels of cisplatin were detected inside the tissue of tumors pre-treated with RAPTA-T compared to tumors that received no pre-treatment (Fig. 6C), thus demonstrating increased drug uptake into tumors with improved vascular function and supporting the hypothesis that changes to the vasculature induced by RAPTA-T leads to a better distribution of the cytotoxic drug. We further confirmed the beneficial vascular transport conditions created by RAPTA-T in an orthotopic mesothelioma model by pre-treating with RAPTA-T followed by lipoplatin, and demonstrated improved tumor regression for a longer period of time in tumors pre-treated with RAPTA-T compared to tumors that did not receive the pre-treatment (Fig. 7).

The limited efficacy of cisplatin for mesothelioma is potentially due to limited amounts of the drug that enters the tumor. The typical high interstitial pressure in mesothelioma can be a major barrier to drug delivery and distribution, especially for macromolecular drugs, which rely on convection rather than diffusion for their tumor penetration. RAPTA-T caused a significant decrease in tumor IFP in our study which was associated to enhanced vascular pericyte coverage (vessel stabilization). The latter would result in decreased permeability, enhanced convection and drug distribution. Alternative effects of RAPTA-T could be related to an improved overall vascular perfusion that is suggested by the increased vascular density assessed by intravital microscopy and the decreased hypoxemic tumor regions (based on the pimonidazol immunostainings). Cisplatin and lipoplatin differ significantly in size, however the potential binding of cisplatin to the plasma protein albumin suggests a convective transport for the small molecule drug^28^. We observed enhanced distribution of both with RAPTA-T treatment. Vessel stabilization combined with enhanced perfusion (and oxygenation) are likely the contributing factors that helped to enhance drug distribution for both cisplatin and lipoplatin. Some studies have suggested that extracellular matrix content could affect regional blood perfusion through shear stress^27^. However, although tumor associated fibroblasts (alpha-SMA positive cells not associated to vessels) increased with RAPTA-T, our extracellular matrix analysis failed to show a difference in RAPTA-T treated tumors compared to controls regarding collagen and hyaluronic acid content. The increased uptake of the chemotherapeutic drug through pre-treatment with RAPTA-T significantly improves the treatment outcome of mesothelioma and potentially other cancer types. In addition, the enhanced oxygenation within tumors is also a predictor of better response of tumors to radiation therapy, as previously shown with anti-angiogenic therapy, and suggestive of a better response of these tumors to radiation therapy.

## Methods

### Compounds

RAPTA-T was prepared as previously described^17^.Cisplatin was obtained from TCI (Zwjndrecht, Belgium) and liposomal cisplatin (Lipoplatin) was purchased from Regulon (Athens, Greece). FITC-dextran (2,000 kDa) was purchased from Sigma-Aldrich (Buchs, Switzerland).

### Tumor cell lines and cell culture

The human mesothelioma MTSO-211H cell line was obtained from the American Type Culture Collection (American Type Culture Collection LGC Standards GmbH, Wesel, Germany) and H-Meso-1 from CLS Cell Lines Service GmbH (Eppelheim, Germany). Both were cultured in RPMI 1640 medium supplemented with HEPES (Thermo Fisher Scientific, Reinach, Switzerland) and 10% fetal bovine serum (PAN Biotech, Eidenbach, Germany). The H-Meso-1 cell line was transfected with a luciferase lentiviral construct (pGL3) as described previously (H-Meso-1-lucP,^29^. The H-Meso-1-lucP cell line was routinely passaged with 2 µgmL^−1^ puromycin as selection antibiotic in the cell culture medium to ensure the stable expression of luciferase in this cell line. Cells were grown in a humidified atmosphere with 5% CO_2_ at 37 °C and regularly checked to be free of Mycoplasma contamination.

### Animals

All animal experiments were approved by the Institutional Animal Care and Use Committee of the University of Lausanne (Authorization VD2923). All procedures were carried out in accordance with the approved guidelines. Six-week old female Swiss nude mice were purchased from Charles River (Lyon, France). All animals were kept in a specific pathogen free environment, which included filtered air, sterilized food, water, bedding and cages until their visualization. All surgeries and imaging of the dorsal skinfold chamber were performed under ketamine/xylazine anesthesia to avoid any hypotension.

### Dorsal Skinfold Chamber Model

MSTO-211H human mesothelioma cells (5 × 10^5^ cells) were inoculated in serum-free RPMI 1640 medium in the subcutaneous dorsum of nude mice. Tumor growth was assessed on a daily basis. When tumors reached a size of 3–4 mm in diameter (between 2 and 3 weeks), a custom built dorsal skinfold chamber was implanted on the back of the mouse, as previously described^29^. Briefly, mice were anesthetized and kept warm using a heating pad throughout the surgical procedure. A skinfold was created on the dorsum of the mouse to obtain the tumor on one side and clear skin on the other. The layer of skin on the back of the tumor was removed surgically in order to fit the glass coverslip. Once completed, this model allowed a visual access to the vascular network of tumor and surrounding tissue over multiple days with no further surgical re-intervention.

### Fluorescence Intravital Microscopy

Dorsal skinfold chambers were imaged using a fluorescent upright microscope Carl Zeiss Axiotech Vario 100 microscope equipped with a Hg-arc lamp (OSRAM GmbH, Augsburg, Germany) filtered for excitation at 470 ± 20 nm (Carl Zeiss “cube filter set 09”, exc BO 450–490, DM FT510, em LP515) and powered through a variable Carl Zeiss FluoArc device that allowed to dim the light power in order to prevent excessive excitation and photobleaching during imaging. Achroplan Carl Zeiss 2.5 x /0.0075 and a 4 x /0.10 Plan Neofluar objectives were used for a large field view (3 × 3 mm^2^ for the 4 x objective) and a 20 x /0.50 water immersion objective was used for close observation of the capillaries (field of view 600 × 600 µm^2^). The mouse was placed in a lateral decubitus position inside a plexiglas tube that was positioned under the microscope. The chamber was fixed horizontally which allowed performing transillumination and epi-illumination with no change in animal position (Fig. S3). Real-time images were recorded by a CCD camera (EM-CCD C9100-12, 400–1000 nm, Hamamatsu Photonics, Solothurn, Switzerland) using an exposure time of 500 ms and a gain factor of 10. The Hamamatsu HiPic version 7.0 software produced 16-bit grey level images with a size of 512 × 512 pixels. Prior to imaging, a fluorescent dye was injected intravenously (100 µL of sterile-filtered FITC-dextran 2,000 kDa (10 mgmL^−1^) in 0.9% NaCl solution into the lateral tail vein. Real-time imaging was started after a circulation time of 5 min and followed over a period of 30 min with an image acquisition every 10 min. Each mouse was imaged every other day for up to 21 days post surgery.

### Image Analysis

Image analysis was performed using the ImageJ/Fiji 1.5.1 software (Wayne Rasband, NIH, USA). Total vessel density in dorsal skinfold chambers was assessed using the DeBacker method^30^. Briefly, a minimum of three 91 × 91 pixels regions of interest (ROI) excluding necrotic areas was randomly selected on each image (*N* = 4 for controls and *N* = 3 for RAPTA-T treated). On each ROI, three equidistant horizontal and three equidistant vertical lines were drawn. The number of vessels crossing the lines was counted manually and vessel density was reported as the DeBacker score index (number of vessels crossing the lines divided by the total length [mm] of the lines). These results were then validated on tumor sections stained for CD31 by quantifying the area of CD31 positive vessels. In addition, mean vessel diameters on these sections were quantified by measuring manually the largest distance within cross-sectioned vessels. For each tumor 10 different fields (approx. 300 × 300 µm) were randomly chosen for analysis. Fractions of hypoxic tissue were assessed with the IHC tool of ImageJ/Fiji and were defined as the area fraction of the tissue showing positive pimonidazole staining.

### Heterotopic Tumor Model and Treatment Protocol

Tumors were grown by subcutaneous injection of 1 × 10^6^ MSTO-211H mesothelioma cells in 100 µL of serum-free RPMI 1640 medium on the flank of seven-week-old mice. Treatment was started after 4–5 weeks when tumors reached a volume of 100 mm^3^. Animals were randomly divided into four groups (*N* = 5 per group). (I) Control Group: one intraperitoneal (i.p.) injection of 100 µL of 0.9% NaCl (aq.) per day for 10 consecutive days. (II) RAPTA-T Group: 50 mg kg^−1^ of RAPTA-T in 0.9% NaCl (aq.) (total volume 100 µL) i.p. per day for 10 consecutive days. (III) Combined RAPTA-T/cisplatin group: 50 mg kg^−1^ of RAPTA-T i.p. per day for 10 consecutive days followed by i.p. administration of 4 mg kg^−1^ of cisplatin in 0.9% NaCl (aq.) on day 11. (IV) Cisplatin control group: 100 µL of l 0.9% NaCl (aq.) i.p. each day for 10 consecutive days followed by 4 mg kg^−1^ i.p. injection of cisplatin in 0.9% NaCl (aq.) on day 11. The tumor volume was measured with a digital caliper every 2 days and calculated using the modified ellipsoid formula V = (L × l^2^)/2, where L is the widest and l the smallest diameter.

### Immunohistochemistry and Immunofluorescence

To obtain tissue sections for pericyte coverage assessment, tissues were fixed by transcardial perfusion of 10% neutral buffered formalin solution at a controlled pressure of 70 mmHg applied right after animal death induced by pentobarbital injection. Tumors were harvested, fixed in 10% neutral buffered formalin solution for 24 h, immersed in 30% sucrose solution for 48 h, washed in PBS and embedded in paraffin or frozen in cryo-embedding medium (OCT Bioptica, Milan Italy). For CD31 staining, 8 µm thick paraffin sections were used. Tumor sections were deparaffinized, rehydrated and antigen retrieval was performed in 10 mM citrate buffer (pH 6.0) for 20 min at 95 °C. Endogenous peroxidase activity was inhibited by incubation with 3% H_2_O_2_ for 10 min in the dark. After blocking nonspecific reactivity with 1% BSA for 30 min, samples were incubated overnight 4 °C with rat anti-CD31 antibody (1:100, DIA-310-M, Dianova, Hamburg, Germany), followed by ImmPRESS HRP anti-rat IgG secondary antibody (Vector Laboratories, Burlingame, USA) for 30 min at room temperature. The immunoreaction was developed using diaminobenzidine peroxide solution. Cell nuclei were counterstained with hematoxylin. Double-immunofluorescence staining of CD31 and α-SMA was performed on 8 µm thick frozen sections. Endothelial cells were stained by anti-CD31 as described above. Pericytes were stained by anti-α-SMA (1:100, 1A4, Dako, Glostrup, Denmark) antibody for 40 min at room temperature, followed by secondary antibodies donkey anti-mouse Alexa488 (1:800) and goat anti-rat Alexa568 (1:1000). To detect tumor hypoxia, 60 mg kg^−1^ pimonidazole was injected intravenously 1 h before animals were sacrificed. Tumors were rapidly resected and frozen at −80 °C. A Hypoxyprobe-Kit (Chemicon, Burlington, MA, USA) was used to detect pimonidazole-protein adducts following the manufacturer’s protocol. All quantitative studies were carried out on preparations cut sagittally through the central regions of tumors.

### Pericyte Coverage Assessment

All sections were scanned by an epifluorescent Olympus microscope equipped with an Olympus XM10 B/W camera and an Olympus Slide Scanner VS120-L100 (all performed with a 20x objective). To estimate pericyte coverage, the overlap between α-SMA+ signal and CD31+ signal in MSTO-211H tumors (3 mice per group with 15 different 300 × 300 µm size fields per section). The BIOP Threshold Finder plugin of ImageJ/Fiji was used to determine the best threshold levels for the CD31 and α-SMA signals, which were then applied to all images. The Colocalization plugin function of ImageJ/Fiji was used to identify the number of overlapping pixels from the thresholded images. The amount of colocalization was expressed as the overlapping pixels divided by the total CD31+ pixels from the thresholded images. Cancer-associated fibroblasts were considered present if α-SMA immunoreactivity was visible anywhere away from the vessel perimeter. To estimate the number of cancer-associated fibroblasts, α-SMA-positive cells which are not associated to endothelial cells were counted on the same tumor sections using ImageJ/Fiji.

### Quantification of Cisplatin and Ruthenium Drug Uptake in Tumors

Ruthenium and platinum content in explanted MSTO-211H tumors from different treatment groups was assessed by Inductively Coupled Plasma Mass Spectrometry (ICP-MS). The tumor samples were digested with 400 µL of 69% HNO_3_ for 24 h at room temperature and adjusted with ultrapure water to a final volume of 4 mL. Indium was added as an internal standard at a concentration of 1ppb. External standards were prepared gravimetrically in an identical matrix to the samples (with regard to internal standard and nitric acid) with single element standards. Ru, Pt and In standard solutions (1gL^−1^ in 2% HNO_3_, 2% HNO_3_ and 10% HCl, respectively) were purchased from CPI International (Amsterdam, The Netherlands). Determinations of total metal contents were achieved on an Elan DRC II ICP-MS instrument (Perkin Elmer, Switzerland) equipped with a Meinhard nebulizer and a cyclonic spray chamber.

### Interstitial Fluid Pressure Determination

IFP was measured in the center of tumors using the wick-in-needle-technique^12,31^. A custom-made 25-gauge needle with a 1 mm side hole located approximately 2 mm from the needle tip was coupled to a pressure sensor by a water column in polyethylene tubing (0.54 mm inner diameter), filled with heparinized water (70 UmL^−1^). Two nylon sutures (6-0) were threaded through the needle to form the “wick.” The signal from the pressure sensor was passed through an amplifier and digitalized in a MacLab/4e AD Instrument Corporation (Dunedin, New Zealand) converter. Data were collected using the PowerLab Chart software version 4.2 (AD Instruments Ltd). Care was taken to exclude air bubbles from the entire system. Before each experiment, the system was calibrated against a defined height by placing the needle at the level of insertion (zero reference) and at a defined elevation of 30 cm. Following anesthesia, one needle was then introduced at the center of the tumor and another calibrated needle was placed parallel under the normal skin 5 mm apart and at the same height of the tumor. Fluid communication between the tumor or subcutaneous tissue and the pressure transducer was checked by briefly clamping the tubing, hence causing a brief compression and decompression of the tube; when fluid communication was satisfactory, IFP quickly returned to the same value as before the clamping operation. Changes of interstitial fluid pressure were determined at three timepoints (day 0, 4 and 8) following control or RAPTA treatment over a total time course of 8 days. Three independent measurements were taken where IFP values for each measurement were followed over a period of at least 15 min with brief clamping every 5 min. The values after clamping were allowed to stabilize and give the mean IFP per measurement. After each measurement, the needle was removed and calibration was checked against the 0 and the 30 cm value to ensure no clogging had occurred and that fluid communication was free. For each tumor the change in IFP over time is reported by subtracting the initial tumor IFP at day 0 from the IFP of each time point (day 0, 4 and 8). One animal in the control group (*N* = 7) showed a continuous decrease in IFP over 8 days and two animals in the treatment group (*N* = 9) had an increase in IFP over 8 days and were excluded as outliers from the analysis.

### Orthotopic Tumor Xenograft Model and Treatment Protocol

Mice were anaesthetized with ketamine/xylazine and placed in position of the right lateral decubitus. A 29 gauge needle of a 500 μL syringe was advanced through the fourth intercostal space for about 5 mm, into the left pleural cavity, and 10^6^ H-MESO-1-lucP cells suspended in 100 μL of PBS were injected. Treatment was started 3weeks thereafter, when tumors reached a volume with a photon flux >2 × 10^5^. Animals were randomly divided into two groups (*N* = 4 per group), and treatment was given in a total volume of 100 μL consisting of (I) Group control: one i.p. injection of 0.9% NaCl (aq.) for 8 consecutive days every 24 h, (II) Group RAPTA-T: one i.p. injection of RAPTA-T (50 mg kg^−1^) in 0.9% NaCl (aq.) for 8 consecutive days every 24 h. A single injection of lipoplatin (18 mg kg^−1^; corresponds to one third of the LD50 in mice) was administered i.p. on day 9 to both groups.

### Bioluminescence Imaging

Tumor sizes were assessed by bioluminescence measurement using the IVIS Imaging System (Caliper Life Sciences, Hanover, MD) as previously described. Briefly, 100 μL of D-luciferin (30 mgmL^−1^, Promega, Dübendorf, Switzerland) were injected i.p. 15 min prior imaging. During the entire course of monitoring, the animals were stilled by isoflurane anesthesia. The tumor sites showing bioluminescence were identified as the regions of interest (ROI) and the total photon counts were quantified using the software Living Image 3.1.0 (Caliper Life Sciences). The tumor volume (or ROI) was measured by bioluminescence imaging two times per week over a period of 29 days and was determined by subtracting the bioluminescence signal of a control mouse with no implanted tumor. The tumor volume in each group was expressed as the average values of the total photon counts.

### Statistical Analysis

Values are given as mean values ± standard deviation (SD) if not otherwise stated. Statistical analysis was performed with GraphPad Prism software (GraphPad Software Inc., CA). Statistical tests, group sizes and *P* values are given in the corresponding figure legends. *P* values < 0.05 were considered statistically significant.

### Data Availability

The datasets generated during and analyzed during the current study are available from the corresponding author.

## Electronic supplementary material

Supplementary material
