# Supplementary material for: Chemo-manipulation of tumor blood vessels by a metal-based anticancer complex enhances antitumor therapy
Source: Sci Rep. 2018 Jul 6;8:10263. doi: 10.1038/s41598-018-28589-2 (PMC6035176; doi:10.1038/s41598-018-28589-2)
Supplement: Supplementary file 1 — Supplementary material [file 41598_2018_28589_MOESM1_ESM.docx]

Chemo-manipulation of tumor blood vessels by a metal-based anticancer complex enhances antitumor therapy

**Authors**

Tina Riedel,^1^ Sabrina Cavin,^2^ Hubert van den Bergh,^1^ Thorsten Krueger,^2^ Lucas Liaudet, ^3^ Hans-Beat Ris,^2^ Paul J. Dyson,^1^ Jean Y. Perentes^2^

**Supplementary material**

Supplementary methods:

**PARP-1 activity determination in vitro**

PARP-1 activity was determined by using Trevigen’s HT Universal Colorimetric PARP Assay kit (cat # 4677-096-K). This assay measures the incorporation of biotinylated poly(ADP-ribose) onto histone proteins in a 96-well strip well format. Purified human high specific activity PARP1 (PARP-HSA) enzyme was incubated with serial dilutions of RAPTA-T ranging from 10 to 1000 µM for 24 hours before assessing its activity. The incubation time was chosen according to previous published studies[25, 31]. The ribosylation reaction and the detection was performed according to the protocol supplied by the manufacturer.

**Evaluation of PARP-1 inhibition in MSTO211H cells**

*MSTO211H cells treatment*

Cells were seeded at 1х10^5^ cells/well in 24-well plates for cell viability measurement or at 5х10^6^ cells/dish in 100 mm cell culture dishes to assess poly(ADP-ribose) (PAR) content in cell lysates by western blot analysis. Subconfluent cell cultures were serum starved and pre-incubated or not with 400 µM of RAPTA-T for 24 hours. The cells were then treated with H_2_O_2_ at 250 uM for 30 minutes for detection of PAR formation by western blotting or 24 hours for cell viability measurement. 3-aminobenzamide (3-AB) at a concentration of 150 μM was used as a positive control for PARP-1 inhibition and was added into cell culture vessels 1 hour before H_2_O_2_.

*Cell viability measurement*

Cell viability was assessed by using PrestoBlue^TM^ cell viability reagent (Invitrogen). Cells were incubated with PrestoBlue^TM^ for 2 hours and fluorescence was measured with an Infinite 200 PRO TECAN plate reader with excitation at 560 nm and emission at 590 nm.

*Western blotting*

Cells were lysed in RIPA buffer supplemented with protease inhibitor cocktail (Complete, EDTA-free, Roche). Samples containing 25 μg of proteins were denatured in SDS-PAGE sample buffer and separated on 10% acrylamide gels and electroblotted onto nitrocellulose membranes. The blots were incubated with a mouse anti-poly(ADP-ribose) polymer (PAR) antibody (Abcam, ab14459, 1:1000 dilution) and an anti-α-tubulin monoclonal antibody in combination with an anti-mouse HRP secondary antibody (Promega, W402B, 1 :20000 dilution).

PAR and α-tubulin signals were detected by using Amersham ECL western blotting detection reagent (GE Healthcare Life Sciences).

**Measurement of VEGF**

Cells were serum starved and incubated or not with 400 µM of RAPTA-T for 24 hours. Cell culture supernatants were collected and clarified. The amount of VEGF in the supernatants was evaluated by using VEGF human ELISA kit (Thermo Fisher Scientific, BMS277/2) according to the protocol supplied by the manufacturer.

**Immunohistochemistry and Immunofluorescence**

(To obtain tissue sections, tissues were fixed by transcardial perfusion of 10% neutral buffered formalin solution at a controlled pressure of 70 mmHg applied right after animal death induced by pentobarbital injection. Tumors were harvested, fixed in 10% neutral buffered formalin solution for 24 h, immersed in 30% sucrose solution for 48 h, washed in PBS and embedded in paraffin or frozen in cryoembedding medium (OCT Bioptica, Milan Italy).

For collagen I and hyaluronic acid staining 4 μm thick paraffin sections were used. Tumor sections were deparaffinized, rehydrated and antigen retrieval was performed in 10 mM citrate buffer (pH 6.0) for 20 min at 95°C. After blocking non specific reactivity with 1% BSA for 30 min, samples were incubated overnight at 4°C with rabbit anti-collagen I antibody (1 :100, Abcam, ab34710) or with Hyaluronic Acid Binding Protein (HABP) biotin (1 :100, Millipore, 385911) followed respectively by donkey anti rabbit Alexa 568 (1 :1000, Molecular Probes) or streptavidin Alexa 568 (1 :800, Molecular Probes) for 40 min at room temperature.

Triple immunofluorescence staining of poly(ADP-ribose) polymer (PAR), CD31 and α-SMA was performed on 4 µm thick paraffin sections. Tumor sections were deparffinized, rehydrated and incubated with 3% H_2_O_2_ for 10 minutes in the dark to inhibit endogenous peroxidase activity. Antigen retrieval was performed and non specific reactivity was blocked as described above. Samples were then incubated 1 hour with mouse anti-PAR antibody (1 :400, Abcam, ab14459) and the signal was amplified and revealed by using the Tyramide Signal Amplification technology **(**TSA™, secondary antibody : ImmPRESS™ HRP anti-mouse IgG secondary antibody (Vector Laboratories, Burlingame, USA) for 30 min at room temperature followed by 10 minutes incubation with TSA-Alexa488). Tumor sections were briefly boiled in 10 mM citrate buffer (pH 6.0) and incubated overnight at 4°C with mouse anti- α-SMA (1 :100, clone 1A4, DAKO) and rat anti-CD31 (1 :50, Dianova, DIA-310-M) antibodies followed by anti mouse Alexa 568 and anti rat Alexa 647 secondary antibodies for 40 min at room temperature.

**Image Analysis**

Image analysis was performed using the ImageJ/Fiji 1.50 software (Wayne Rasband, NIH, USA). For the evaluation of collagen I and hyaluronic acid content within tumors, tumor sections were scanned with a Zeiss Axioscan Z1 slide scanner at a 20x magnification. A threshold value of 100 was applied for collagen I and a threshold value of 70 was applied for hyaluronic acid. For each tumor, the percentage of collagen I and hyaluronic acid was obtained by dividing the collagen I and hyaluronic acid corresponding area by the tumor area.

For the analysis of PAR staining around vessels and pericyte coverage in orthotopic tumors, 6 fields per tumor were acquired with a Zeiss LSM 880 inverted confocal microscope as a 2×2 tile using four 512×512 pixels at a 40× magnification. The tiled images were then stitched with the Zen black zeiss software to minimize discontinuities.

To assess the number of PAR + nuclei around vessels, a region of interest (ROI) whose borders are located 100 μm far from tumor vessel walls (CD31+ pixels) was delimited. To that end, a threshold value of 90 was empirically determined to select CD31+ pixels and applied to all images. The selected pixels were then dilated 50 times consecutively to define the ROI. A threshold value of 60 was empirically determined for the PAR signal and applied to all images. The number of PAR+ particles with a size of 30 to infinity microns^2^ into the ROI was then measured and normalized to the total amount of vessels per field (CD31+ pixels) to sample the tumor PAR expression only (greater nuclei size compared to endothelial or perivascular cells).

To assess pericyte coverage of orthotopic tumors vessels, the best threshold levels for the CD31 (90) and α-SMA (60) signals were empirically determined and applied to all images. The Colocalization plugin function of ImageJ/Fiji was used to identify the number of overlapping pixels from the thresholded images. The amount of colocalization was expressed as the overlapping pixels divided by the total CD31+ pixels from the thresholded images.

Supplementary Figure Legends

Supplementary Fig 1:

A: Inhibitory effect of increasing concentrations of RAPTA-T (10-1000 μM) on PARP-1 activity was determined. The IC_50_ of RAPTA-T was around 400 μM. Data are represented as mean ± SD (N=3/group).

B: Cell viability assay in the presence of H_2_O_2_ with or without RAPTA-T and the prototypical PARP-1 inhibitor 3-AB. At a concentration of 400 μM, RAPTA-T improved tumor cell survival in the presence of H_2_O_2_ in a way comparable to 150 µM of 3-AB, consistent with the activity of RAPTA-T as a PARP-1 inhibitor. Data are expressed as meand ± SD (N=4/group). *:*p*<0.05, 1-way ANOVA

C. PARP-1-mediated PAR polymer formation was assessed by western blotting on MSTO211H cell lysates, previously exposed or not to 250 μM of H_2_O_2_, with or without RAPTA-T (400 µM) or 3-AB (150 μM).The quantification of the PAR western blot is shown as the fold increase in PAR polymer formation compared to the RAPTA-T treated group. Formation of PAR polymers tended to be reduced by RAPTA-T and 3-AB, in agreement with their PARP-1 inhibitory activity. Data are represented as mean ± SD (N=3/group).

D: VEGF-A levels in MSTO211H tumor cell conditioned media treated with RAPTA-T (400uM) or untreated (controls). VEGF significantly decreased in the presnec of RAPTA-T. Data are represented as mean ± SD (N=3/ group). **P*<0.05, unpaired t-test.

Supplementary Fig 2:

Effect of Rapta-T on cell viability of mesothelioma MSTO211H and Hmeso1 cells. Cells were treated with RAPA-T for 72H and cell viability was measured by calorimetric endpoint. Data represent mean ± SD for each triplicate. Mean IC50 values are 446 ± 16µM for MSTO211H and 357 ± 5 µM

Supplementary Fig 3:

Real time assessement of MSTO211H tumor and vascular changes following different concentrations of RAPTA-T therapy. Images of tumors in dorsal skinfold chambers were taken at indicated timepoints after RAPTA-T treatment at different concentrations (100 µg/kg, 75 µg/kg and 50 µg/kg daily i.p injections). The extent of vessel pruning is dependant on the duration and concentration of the treatment: extensive vessel pruning is observed with 100 µg/kg after 8 days, whereas similar effects were observed with 50 µg/kg of RAPTA-T after 18 days.

Supplementary Fig 4:

A: Collagen I and hyaluronic acid content assessed by immunostaining in orthotopic H-Meso 1 tumors in mice treated with RAPTA-T 50 mg/kg and control mice. No differences in the collagen I or hyaluronic acid content was observed between the two groups.

B: Tumor vascular pericyte coverage in orthotopic H-Meso 1 tumors in RAPTA-T (50 mg/kg) treated-mice or control mice. Pericyte coverage was assessed by alpha-SMA (green) and CD31 (red) double immunostaining for pericytes and endothelial cells respectively.

C: By overlap quantification between the alpha-SMA and CD31 staining, we found that RAPTA-T treated orthotopic tumors have more overlap between pericytes and endothelial cells than controls. Data are represented as mean ± SEM, Control: N=3, 14 fields; RAPTA: N=2, 7 fields.

Supplementary Fig 5:

Pre-treatment with RAPTA-T improves the effect of subsequently applied cisplatin in a heterotopic human mesothelioma xenograft model.

A: Swiss nude mice wer injected s.c. with MSTO211H mesothelioma cells. When tumors reached approx. 100mm3, mice were randomly divided into 4 groups (N=5 per group) and received the treatment regimen described in the scheme.

B: RAPTA-T pre-treatment followed by cisplatin reduced tumor volume by 62 and 51% in comparison to control and cisplatin alone treated groups respectively (p<0.001, Holm-Sidak test).

C: Kaplan meier survival curves reported for the four different treatment groups. The median tumor doubling time for each group is also reported.

Supplementary Fig 6:

RAPTA-T pre-treatment improves the effect of subsequently applied Lipoplatin in an orthotopic Hmeso-1 human mesothelioma xenograft model. The individual tumor growth curves are shown are shown for the RAPTA-T and Control groups (8 daily injections of RAPTA-T 50mg/kg or saline followed by 18 µg/kg of Lipoplatin^®^ injection. Tumor growth and burden was followed by bioluminescence imaging and measured as the total photon flux released by luciferase-expressing H-Meso 1 tumor cells per second. Data represents the average of photon flux/s (log scale) measured from dorsal and ventral positions and given as Mean ± SD).

| Supplementary Figure 1 |
| --- |
| 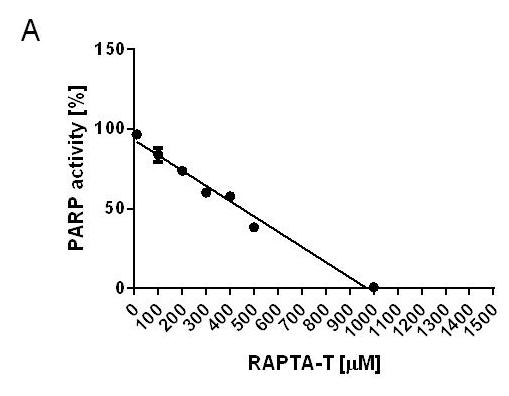 |
| 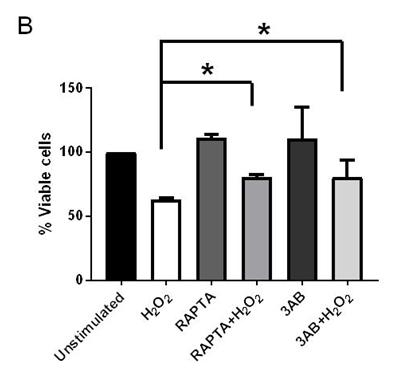 |
| 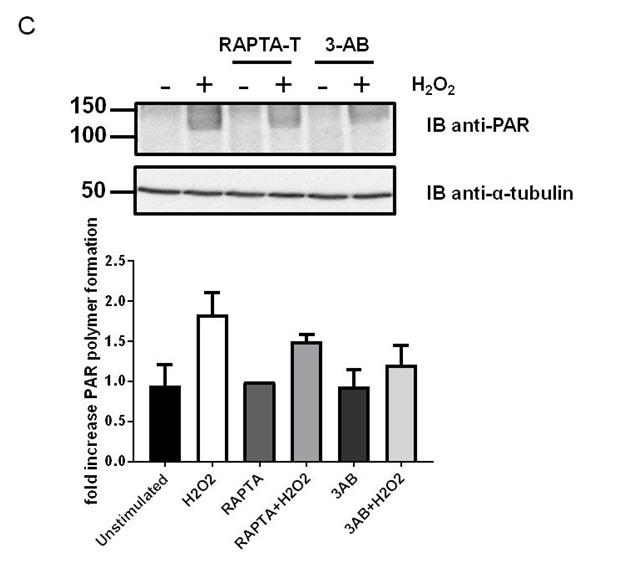 |
| 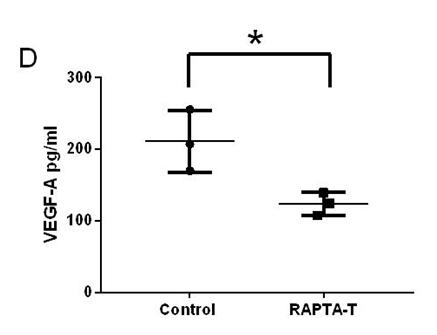 |

Supplementary Figure 2

| 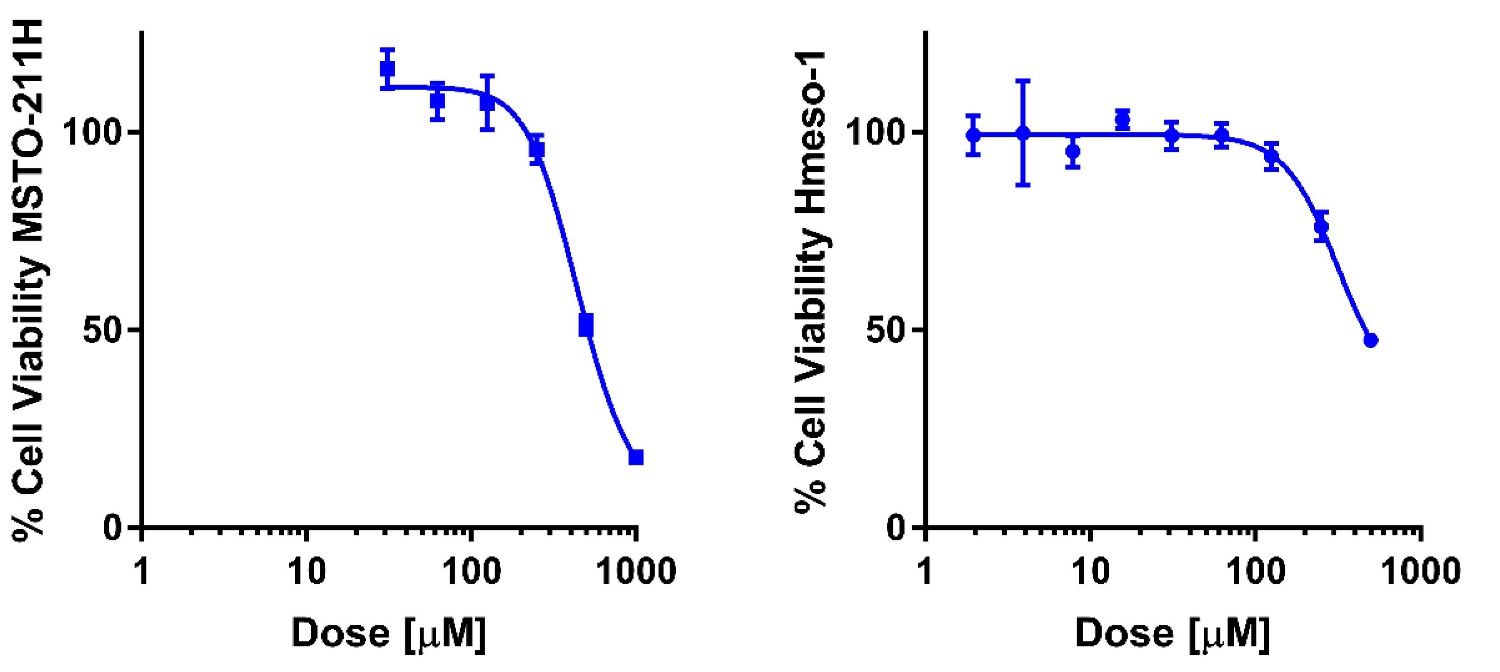 |
| --- |
|  |

Supplementary Figure 3

| 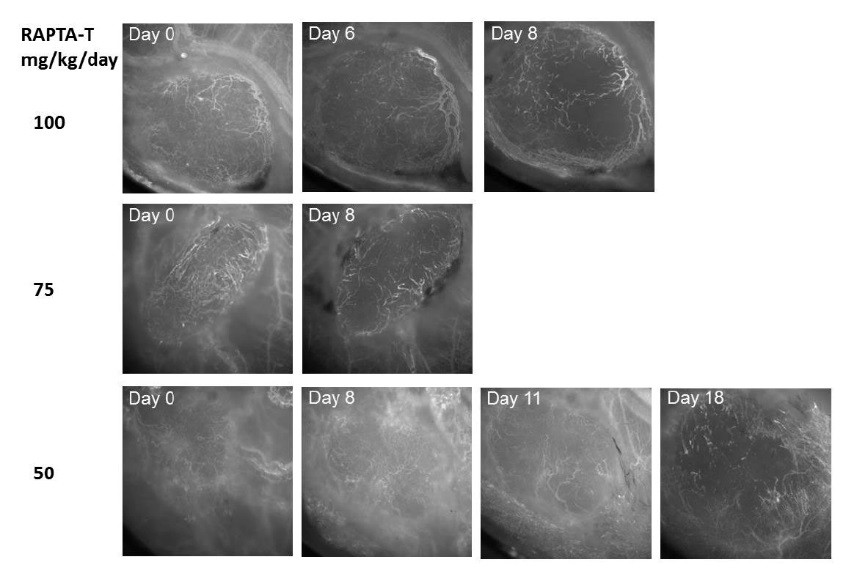 |
| --- |
|  |

| Supplementary Figure 4 |
| --- |
| 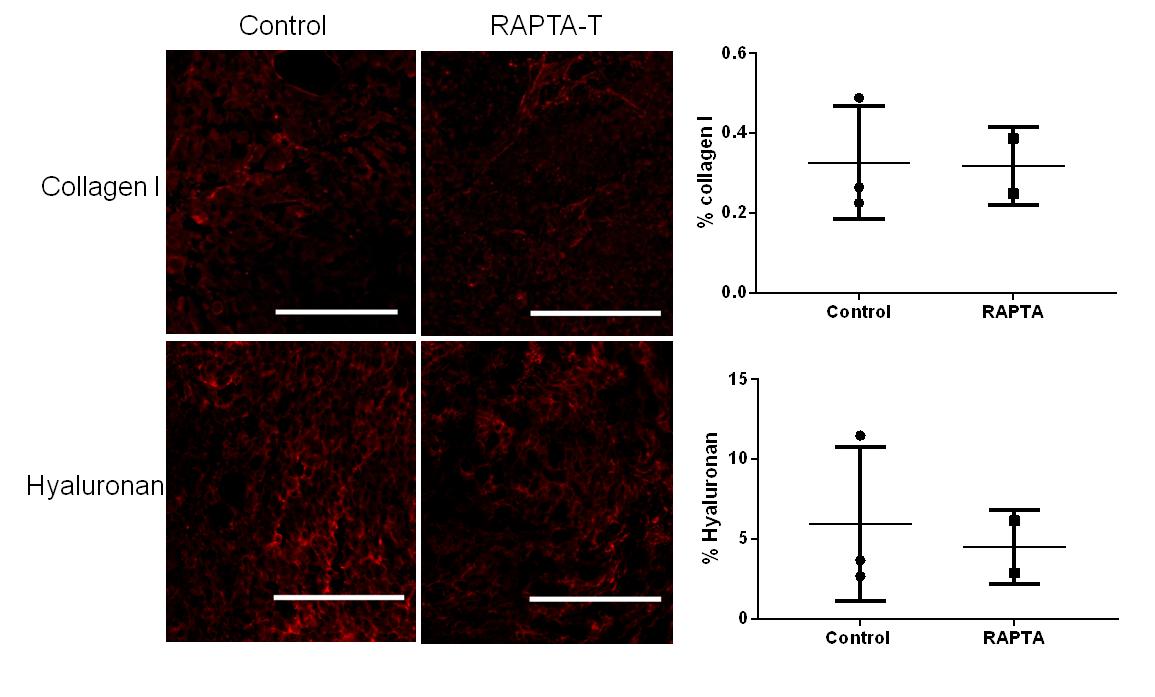  **A** |
| 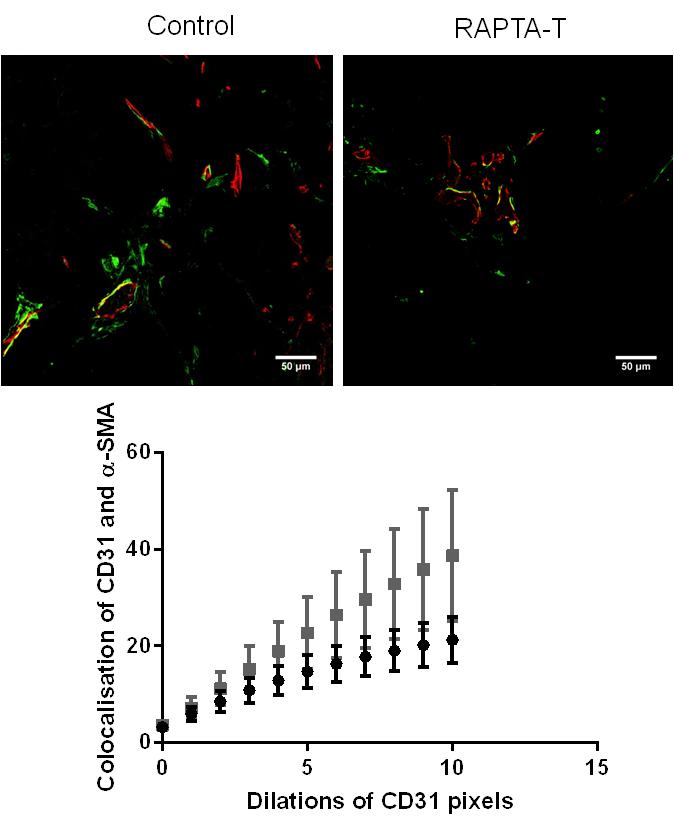  **C**  **D**  **B** |

Supplementary Figure 5

| 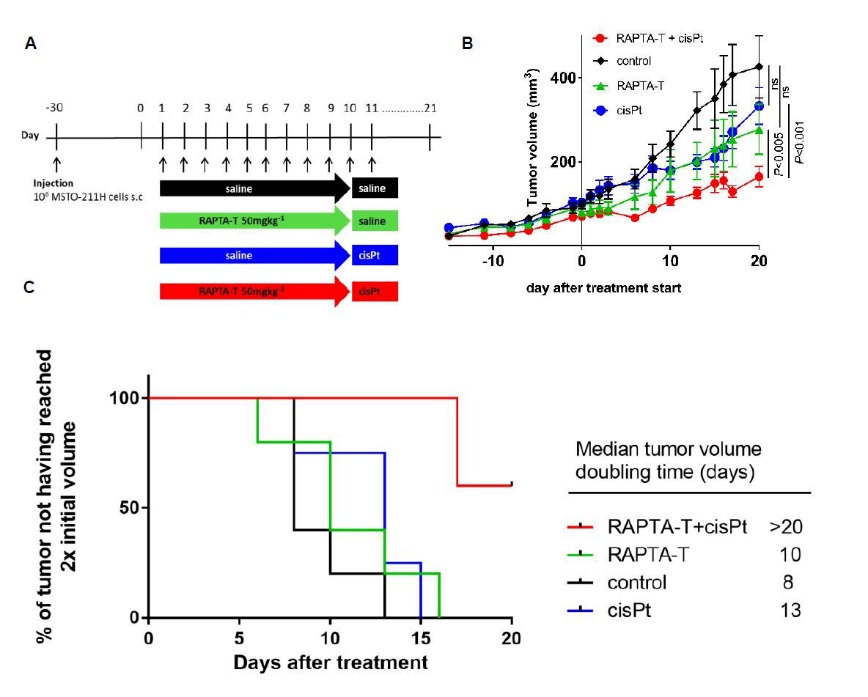 |
| --- |
|  |

Supplementary Figure 6

| 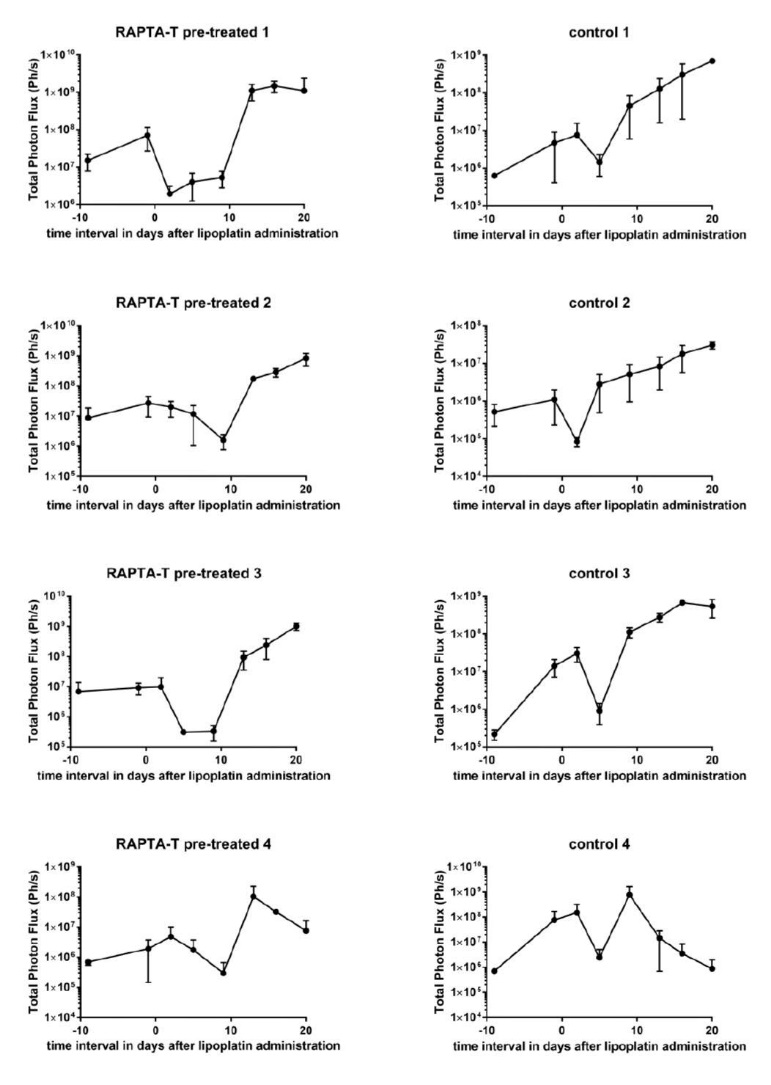 |
| --- |
|  |
